# Calibration Procedure to Test the Effects of Multiple Influence Quantities on Low-Power Voltage Transformers

**DOI:** 10.3390/s20041172

**Published:** 2020-02-20

**Authors:** Alessandro Mingotti, Lorenzo Peretto, Roberto Tinarelli

**Affiliations:** Department of Electrical, Electronic and Information Engineering—Guglielmo Marconi, Alma Mater Studiorum—University of Bologna, Viale del Risorgimento 2, 40136 Bologna, Italy; lorenzo.peretto@unibo.it (L.P.); roberto.tinarelli3@unibo.it (R.T.)

**Keywords:** accuracy, voltage transformer, influence quantities, calibration procedure, temperature, electric field, frequency, ratio error, phase displacement

## Abstract

The article presents a study on low-power voltage transformers (LPVTs). Considering their increasing spread among Smart Grids, it is fundamental to assess their accuracy behavior in as realistic conditions as possible. Therefore, this article presents a detailed calibration procedure to test LPVTs’ accuracy when various external influence quantities are simultaneously acting on them. In the calibration procedure, the considered quantities are frequency, air temperature, and external electric field. Afterwards, the designed procedure is applied on three different off-the-shelf LPVTs using a measurement setup developed in a laboratory environment. The presented results (i) confirm the easy applicability of the designed calibration procedure; (ii) highlight the various effects of the influence quantities on the accuracy of different types of LPVTs; (iii) confirm the need to include more realistic tests, like the type-tests presented, into the standards to appreciate a wider set of possible in-field behaviors.

## 1. Introduction

The world of instrument transformers (Its) has undergone a breakthrough innovation in the last couple of decades. The legacy inductive ITs [1,2,3], which are still largely implemented and relied on by distribution system operators (DSOs) and utilities, are being extensively replaced by a new kind of transformer, known as low-power instrument transformers (LPITs). The LPITs, regulated by the Standards [4,5,6,7], are a completely new way of thinking about ITs. The technology is not limited to the typical inductive type, but a variety of new ways can be implemented to reduce either a current or a voltage. Furthermore, as their name recalls, the LPITs have an output signal with a limited maximum power around 1 voltampere, hence a few volts or milliamperes, that are suitable to direct connectto an acquisition system, avoiding the use of signal adapters that complicate the measurement setup and may decrease its overall accuracy. Finally, another peculiarity of the LPITs is the dimension, which is more compact and reduced compared to legacy inductive ones. This feature of the LPITs is particularly appreciated in a Smart Grid environment where the distributed energy resources, mainly constituting renewable sources, require that the measurement instruments are installed in application where the space for them is limited and not guaranteed.

As for the calibration, several studies are found in literature for ITs and the research is evolving and continuing every day [8,9,10,11,12,13]. In particular, among the studies, three different ways of tackling ITs’ accuracy are distinguished (but not limited to them): (i) the use of compensating techniques to solve the ITs’ nonlinearities; (ii) assessment of the ITs’ behavior when working at off-nominal conditions; and (iii) studies based on preliminary characterization at rated conditions. Instead, the LPITs’ calibration scenario is quite limited, but increasing day-by-day. For example, calibration techniques for electronic transformers are presented in studies by Tong et al. [14] and Cayci and Kefeli [15], while in some studies [16,17,18,19,20] both current and voltage LPITs are tested at rated and off-nominal conditions. Finally, particular attention is given to the calibration of Rogowski coils and low-power current transformers (LPCTs) in other studies [21,22,23,24].

In light of that, this paper consists of a further contribution to the calibration scenario, presenting a simple calibration procedure for low-power voltage transformers (LPVTs). Its peculiarity is that it assesses the accuracy of the transformers when multiple influence quantities are simultaneously affecting the LPVT. The considered quantities are frequency, temperature, and electric field. 

Typically, the effects of external quantities are studied, for ITs and also moderately for LPITs, by considering one quantity at a time. For example, the effect of moisture on current transformers (CTs) and on their position is studied by Yan and colleagues [25] and Ma and co-workers [26], respectively. Arias Velasquez and Mejia Lara [27] and two other studies [28,29] present how CTs and voltage transformers (VTs) behave when subjected to an external electric field and frequency variations, respectively. Lastly, but probably the most important and studied phenomenon, is the effect of temperature on ITs. Such an effect is studied for optical and inductive ITs [30,31,32], for electronic ITs [33], and for CT and LPCTs [34,35,36]. The relevance of the temperature is attributed to the accuracy modification that the device experiences when the operating temperature varies for a change in the ambient conditions or due temperature increases inside the device due to normal operating conditions (or a combination of the two effects).

Turning to the tests combining more than one influence quantity, the authors completed a work on LPCTs [37], in which the accuracy of generic off-the-shelf LPCTs was tested when the simultaneous presence of frequency, temperature, conductor position, and magnetic field was affecting them. In this work, the three quantities are the backbone of the calibration procedure designed to assess the LPVTs’ accuracy when all external quantities are affecting them. Such a calibration procedure is considered a type-test due to its duration which is similar to tests described elsewhere [5,6]. Furthermore, such a procedure is also tested on three off-the-shelf LPVTs of different technologies: resistive, capacitive, and electronic (hence not passive as the others).

There are two predominant reasons for this study: The first is the lack of tests assessing the behavior of LPITs, but in general of ITs, more realistically in field operating conditions. The second is the fact that the measurements performed by the LPITs are collected and sent to intelligent electronic devices, phasor measurement units, etc. [38,39], which run algorithms [40,41,42,43,44] developed and implemented to manage the distribution networks, including control of the protective devices. Therefore, if the accuracy and the measurements obtained with the LPITs are worsened/corrupted by external factors, then all the data processing that follows is affected by the propagation of uncertainty and, in most cases, they become useless or no longer reliable.

What rest of the paper is structured as follows: Section 2 briefly describes the LPVTs using the related standards. The proposed calibration procedure is detailed in Section 3, while in Section 4 all the tests are listed and commented on, and results of the selected case study implementing the procedure are discussed. Finally, Section 5 contains a brief conclusion and some comments on the work and obtained results.

## 2. Low-Power Voltage Transformers 

Before describing the calibration procedure—the aim of this work—it is worth briefly introducing the LPVTs according to the Standards definitions [6,7].

Firstly, an LPIT is an “arrangement, consisting of one or more current or voltage transformer(s) which may be connected to transmitting systems and secondary converters, all intended to transmit a low-power analogue or digital output signal to measuring instruments, meters and protective or control devices or similar apparatus”. In the particular case of measuring LPITs, their output goes directly to measuring instruments.

**Figure 1:**
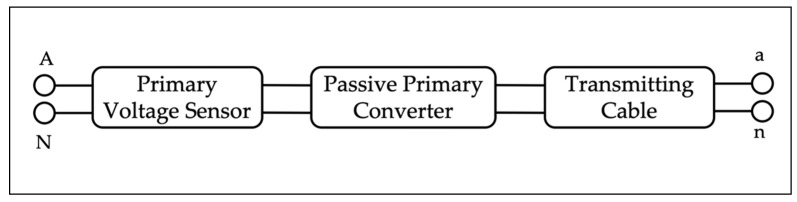
Main structure of a generic passive low-power instrument transformer (LPIT).

Secondly, among the LPVTs—for which the calibration procedure is designed—two main types of devices are distinguished, the passive LPVTs and active ones. A passive LPIT is a “LPIT that includes only passive components” while an electronic LPIT is a “LPIT that includes active components” [4]. As for the former type, its main structure is illustrated in Figure 1; it differs from an electronic LPIT by the presence of a power source and of an active circuit that adjusts the transformers’ output. In the figure, A, N and a, n are the couples of terminals at the LPITs’ input and output, respectively.

As for the LPVTs’ accuracy, which is relevant for this work, Standards [5,6,7] define the ratio error ε and the phase displacement Δφ as:

(1) $$\varepsilon =\frac{KU_{s}-U_{p}}{U_{p}}*100,\;$$

(2) $$\Delta \varphi =\varphi_{s}-\varphi_{p},\;$$

where $U_{p}$ and $U_{s}$ are the rms values of the 50 Hz component of the voltage primary and secondary phasors, respectively; K is the rated transformation ratio of the transformer, and $\varphi_{p}$ and $\varphi_{s}$ are the phases of the 50 Hz components of the voltage primary and secondary phasors, respectively. In addition to Equation (2), the Standards perform a correction of the phase displacement according to the additional information provided by the manufacturers in terms of phase offset to apply.

The accuracy indexes ε and Δφ constitute the backbone for the results evaluation presented in Section 4. 

## 3. Calibration Procedure

In this section, the calibration procedure designed by the authors is presented. The design process started from existing standards and then new type-tests were developed to focus on the effects of multiple influence quantities acting simultaneously on the LPVTs. The influence quantities considered are the frequency, external electrical fields, and temperature. To this purpose, in what follows, for each influence quantity a test is described. Finally, combined tests including more than one influence quantity are proposed and detailed.

The aim of the calibration procedure is to propose a new set of tests to highlight the importance of testing the LPVTs in more realistic conditions (i.e., when a combination of influence quantities is acting on and affecting them). As a matter of fact, in in-field application it is almost impossible to isolate all influence quantities, leaving only one quantity affecting the LPVT. Furthermore, the standards and the literature do not provide any detailed model of the LPVTs to use as starting point for mathematical simulations.

The measurements collected aim at computing ε and Δφ for all the tests included in the calibration procedure.

The uncertainty related to the measurements collected from the defined tests has not been prescribed a rigid method for its evaluation. Such a choice was decided considering that a variety of different equipment and uncertainty evaluation methods might be applied, all leading to the same results. 

### 3.1. Frequency Tests

The frequency test is based on the IEC [4] and consists of computing ε and Δφ at three different frequencies: the rated, the 101%, and the 99% of it. As for the other variables, the voltage of the test is the rated one (typically $20/\sqrt{3}$ kV for medium voltage devices), while the temperature is the room one (24 °C). Finally, a reasonable number of measurements are performed to assess their repeatability and stability over time. It is always difficult to define the meaning of “reasonable”, but 100 measurements is a widely adopted number of measurements that assure a certain repeatability of the performed tests. To this purpose, for this and all the calibration tests that follows, 100 measurements are performed in each test.

### 3.2. External Field Tests

The influence of external electrical fields is typically not given importance in accuracy tests. However, each device is subject to the influence of other phases, considering the three-phase configuration of the network. Therefore, the accuracy vs. external field is important and included in the new standard [6] (for example, the same test is not described for inductive Its in the related standards [1]). 

**Figure 2:**
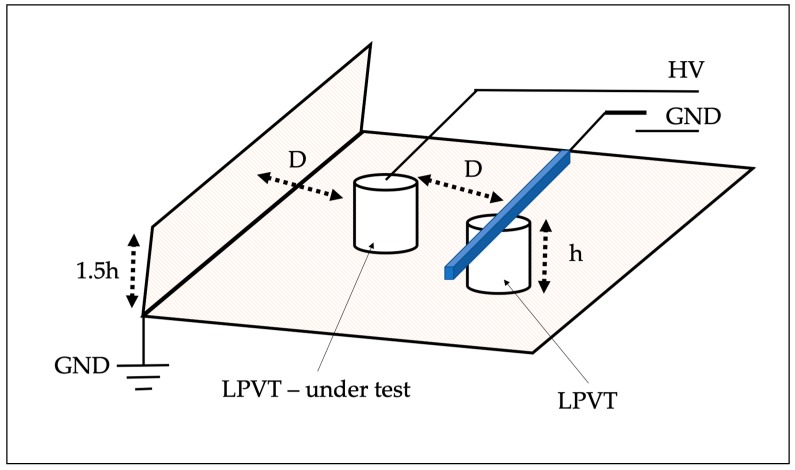
Setup used to test the low-power voltage transformers (LPVTs) when external electrical fields are acting on them.

To perform the test, the setup shown in Figure 2 and defined in [6], needs to be replicated. From the figure, the main elements are highlighted: (i) a base and a vertical wall, both metallic and grounded; (ii) two LPITs, one under testing and the other to replicate the in-field configuration; (iii) a metallic bar installed over the LPIT not under testing, such a bar can be connected to the High Voltage or Ground terminal during the test.

As for the distance D adopted in the picture, the standard [6] does not refer to any length but only to the typical distance between phases in a power system. However, D is used as a measurement unit for the other elements included in Figure 2. In fact, the length of the metallic wall should be at least equal to D, while the height of the wall 1.5 times the height h of the LPVT. Furthermore, D is also the distance between the LPVT under test and the other one, which mounts the metallic bar on the top (which length is D). 

The procedure for this test consists of two steps: Step 1, the metallic bar is grounded, and the rated voltage is applied to the LPVT under testing; therefore, no external electric field effects are affecting the device. Step 2, the metallic bar is connected to high voltage so that both LPVTs are subjected to the rated voltage. In this configuration, the LPVT under test is affected by the electric field generated by the voltage source. For both steps, in addition to ε and Δφ, the actual ratio $k_{a}$ is computed (as the mean of 100 measurements).

Afterwards, Δφ and $k_{a}$ fulfill the requirements if the following conditions are met:

(3) $$k=\frac{k_{a1}-k_{a2}}{k_{a1}}*100\leq \frac{1}{5}\varepsilon_{r},\;$$

(4) $$\varphi_{12}=\Delta \varphi_{1}-\Delta \varphi_{2}\leq \frac{1}{3}\Delta \varphi_{r}.\;$$

In Equations (3) and (4), the subscripts 1 and 2 refer to step 1 and step 2, respectively. As for $\varepsilon_{r}$ and $\Delta \varphi_{r}$ they are the limits of the ratio error and the phase displacement of a generic accuracy class, respectively. For example, in the case of the accuracy class 0.5, $\varepsilon_{r}=0.5\%$ and $\Delta \varphi_{r}=0.006$ rad (see [6] for all the values of each accuracy class).

### 3.3. Temperature Tests

**Figure 3:**
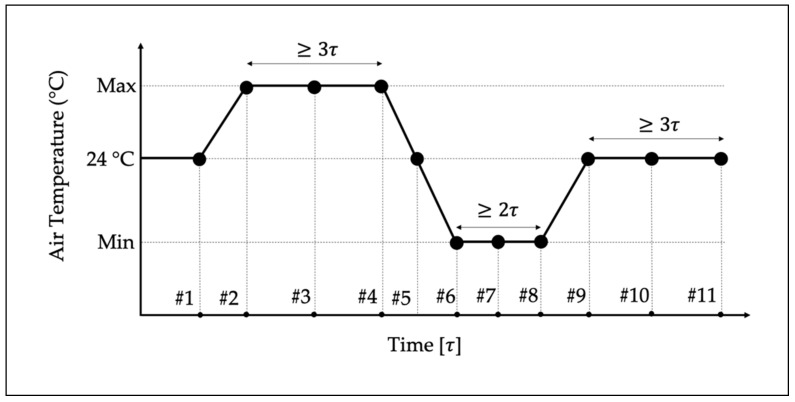
Setup used to test the LPVTs when external electrical fields are acting on them.

The accuracy vs. temperature procedure consists of replicating the thermal curve described in [4] and depicted in Figure 3, when the rated voltage at the rated frequency is applied. The graph contains the air temperature vs. an indication of the duration elapsed of the test according to the LPVT thermal constant τ. Overall, the thermal cycle first consists of an increase in temperature until the maximum temperature allowed, followed by a temperature drop until the minimum value allowed. Finally, the 24 °C temperature is maintained for the last set of measurements. The maximum and minimum values for a LPVT depend on its temperature category, which could be: (i) from −5 to 40 °C; (ii) from −20 to 40 °C; or (iii) from −0 to 40 °C. 

In addition, the graph contains the instant in which the measurements are to be performed (#1 to #11) and ε and Δφ have to be computed. In detail, five measurements are performed at 24 °C, three at the maximum temperature, and three at the minimum temperature. For each of the eleven tests, 100 measurements are performed.

As for τ, it should be provided by the manufacturer, even if is not so common in off-the-shelf products. In the case that such information is not available, the final user should test the LPVT, frequently measuring ε and Δφ until their behavior is considered stable, before moving to the following temperature.

### 3.4. Combined Tests

The core of the calibration procedure consists of the combined tests designed for the LPVTs, all performed at the rated voltage. Such tests are distinguished into two categories: tests with two influence quantities affecting the LPVTs, and tests with all three quantities. 

As for the latter category, the temperature test is replicated as described in Section 3.3 with the difference that, in each measurement point from #1 to #11, 100 measurements are performed:

- For the three frequencies described in Section 3.1 (49.5 Hz, 50 Hz, and 50.5 Hz);
- For the three frequencies when the electric field is acting as described in Section 3.2.

Therefore, from #1 to #11, six tests are performed for an overall of 66 tests aimed at verifying the accuracy performance of the LPVT under test when three influence quantities are affecting it. In other words, for the 66 tests, ε and Δφ are computed 100 times.

Turning to the former category, it aims at testing the LPVTs with a combination of two influence quantities at the time. Therefore, the three tests performed are distinguished as:

- Temperature + electric field. For the 11 measurement points of the thermal cycle, ε and Δφ are computed when the electric field acts on the LPVT (22 tests of 100 measurements each).
- Temperature + frequency. For the 11 measurement points of the thermal cycles, ε and Δφ are computed for the three frequencies of interest (33 tests of 100 measurements each).
- Frequency + electric field. For the three frequencies of interest, ε and Δφ are computed when the electric field is acting on the LPVT (6 tests of 100 measurements each).

The importance of performing all the aforementioned tests lies in the possibility of discovering whether and how the simultaneous presence of more than one influence quantity affects the accuracy parameters of an LPVT. Moreover, the need for such tests is supported by the demand of assessing the LPVTs’ behavior in more realistic conditions.

Confirm of the effectiveness of the presented calibration procedure application is given in the next section, where it is applied on a generic case study involving three off-the-shelf LPVTs.

## 4. Experimental Case Study

### 4.1. Introduction

The calibration procedure described in Section 3 was applied, in a laboratory environment, to three off-the-shelf LPVTs. The aim was to assess the applicability of the proposed calibration procedure and understand how generic LPVTs behave under the simultaneous presence of more than one influence quantity. The selected LPVTs, from here on out, are referred to as A, B, and C for the sake of privacy and because the aim of this work is not to find the better or worst LPVT but to assess their accuracy in the aforementioned conditions. The significant aspect is that A is a resistive LPVT, B is capacitive, and C is an electronic LPVT, as described in the following subsection. 

### 4.2. Measurement Setup

**Figure 4:**
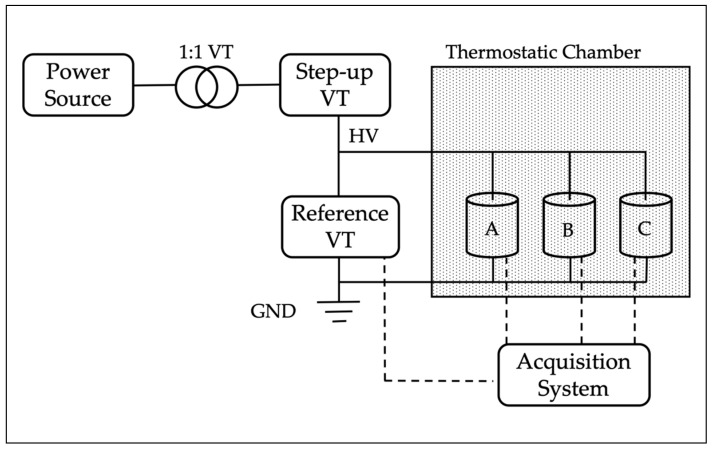
Schematic of the measurement setup adopted for the LPVT testing.

The three LPVTs were tested using the measurement setup depicted in Figure 4. 

It consists of:

- An Agilent 6813B power source. It provided the sinusoidal voltage, at the desired frequency, to the step-up transformer. Between the power source and the step-up transformer an isolating transformer with ratio 1:1 was placed to electrically separate the low and the medium voltage.
- The 0.1/15 kV step-up transformer. It took the low voltage (LV) from the isolating transformer and provided the rated voltage for the LPVTs, which for all of them was $20/\sqrt{3}$ kV.
- A TVM-24-1C reference voltage transformer. It featured a selectable load of 0.25 or 1 VA, an accuracy class of 0.1, and nominal ratio of 20/0.1 kV. The transformer wa under metrological confirmation and was used as a reference for the accuracy performance evaluation of the three LPVTs under test. The transformer was used in series to a 11.0024:1 resistive divider to fit with the acquisition system adopted for the tests. The resistive divider was characterized by means of a Fluke 6105a Calibrator; from the results it emerged that the divider introduces a negligible phase displacement (compared to the quantity measured) and that it has a ratio of 11.0024 with a standard deviation of the mean of $10^{-4}$Ω/Ω.
- A thermostatic chamber. Used for the temperature vs. accuracy tests, it was possible to vary its temperature in the range 5–70 °C.
- An acquisition system consisting of a NI-cDAQ 9174 and one NI-9239 data acquisition board (DAQ). The NI-9239 board is a 24-bit acquisition system with four channels at ±10 V input, 50 kSa/s per channel of maximum acquisition rate, and gain and offset errors of ± 0.03% and ±0.008%, respectively.
- The three LPVTs under test. As mentioned above, they are referred to as A, B, and C and their main characteristics are summarized in Table 1. In particular, the table contains the type of each LPVT, their accuracy class (AC), and their rated primary and secondary voltage values, $V_{1R}$ and $V_{2R}$, respectively. Note, that all LPVTs have the same accuracy class; hence, during the results evaluation a direct comparison is possible. Furthermore, the three LPVTs share very similar $V_{2R}$ that fits with the full scale of the adopted acquisition board.

From a mechanical point of view, the adopted transformers are all cylindrical-shaped, with a height between 23 and 25 cm, and a diameter between 10 and 13 cm. Overall, they are very comparable either from a mechanical or an electrical point of view.

Summarizing the operation of the measurement setup, with the power source it is possible to adjust the output of the step-up voltage transformer (VT) to obtain the test voltage ($20/\sqrt{3}$ kV) at the desired frequency. Then, with the thermostatic chamber it is possible to fix the ambient temperature for the LPVTs and measure their output voltage with the acquisition system in all the conditions described in the calibration procedure of Section 3.

### 4.3. Experimental Tests and Results

#### 4.3.1. Tests vs. Frequency

**Figure 5:**
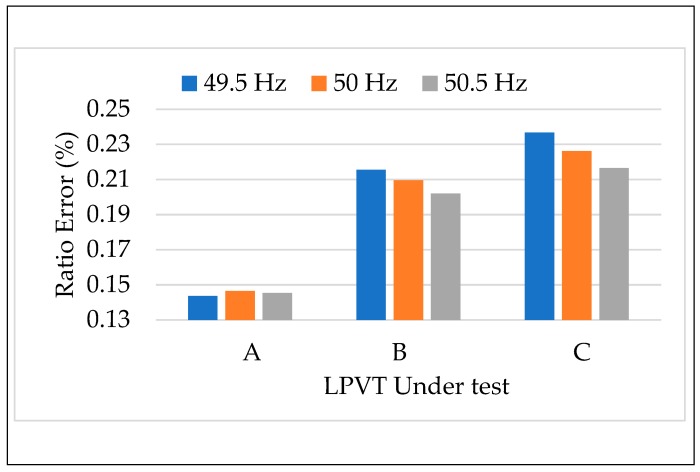
Ratio error vs. frequency for the three LPVTs under test.

**Figure 6:**
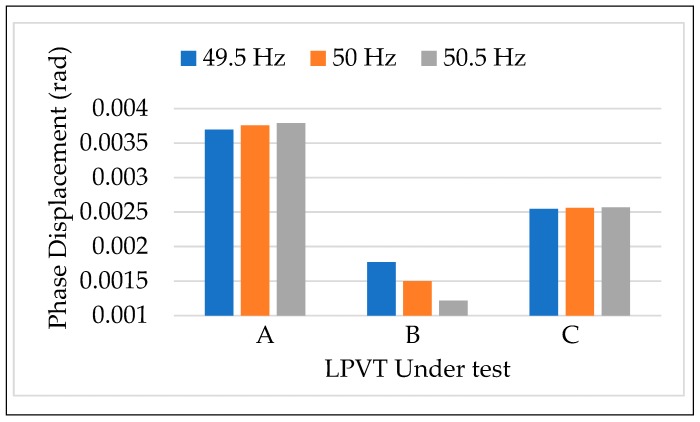
Phase displacement vs. frequency for the three LPVTs under test.

The first test of the calibration procedure applied was the one that involves a frequency variation of the voltage source. Therefore, the rated voltage $20/\sqrt{3}$ was applied to the three LPVTs at the rated frequency 50 Hz and then at 99% (49.5 Hz) and 101% (50.5 Hz) of the rated. In Figure 5 and Figure 6 depict, for the three LPVTs, ε and Δφ obtained as the mean of 100 values. Each frequency is assigned one color that is used throughout the entire manuscript. From these first results, two main comments can be stated. Firstly, at rated conditions (orange bars) and for all the LPVTs, ε and Δφ are largely within the limits of their accuracy class; which are 0.5% and 0.006 rad for ε and Δφ, respectively. Secondly, both accuracy parameters are not influenced by the frequency variation of the primary voltage. Such a result was predictable because it aligned with Standard EN 50160 [45] with the voltage characteristics of the Medium Voltage networks. In fact, the Standard BS EN 50160 [45] fixed the 1 % limit variation of the frequency for 99.5% of the time and the 4–6% limit variation for 100% of the time.

#### 4.3.2. Tests vs. Electric Field

**Figure 7:**
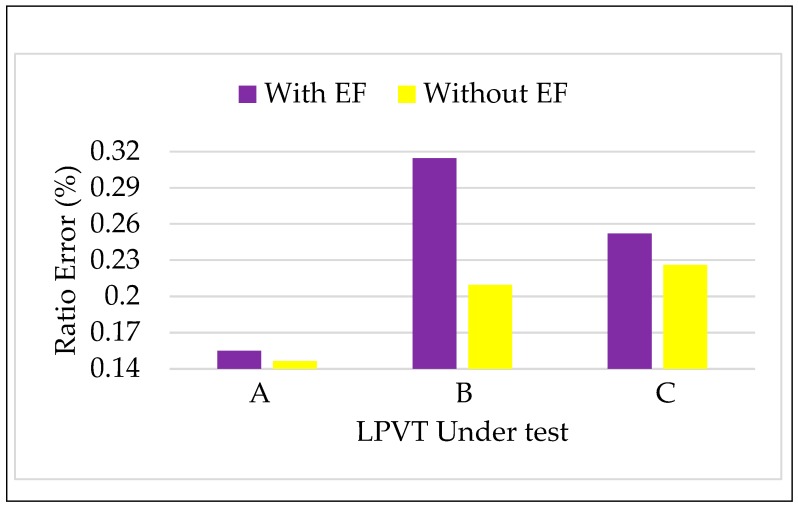
Ratio error vs. electric field for the three LPVTs under test.

**Figure 8:**
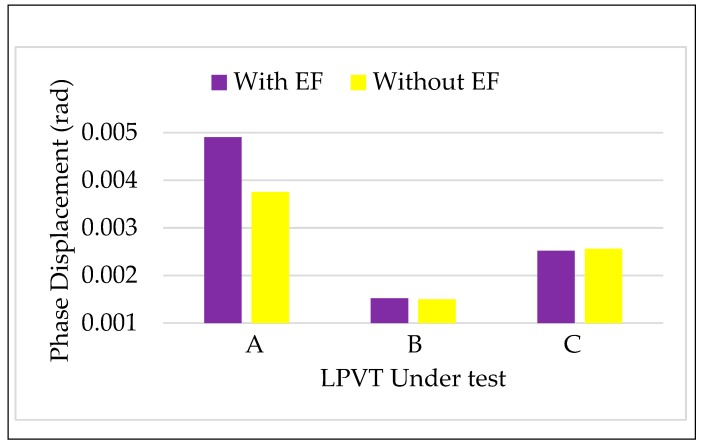
Phase displacement vs. electric field for the three LPVTs under test.

According to Section 3.2, the three LPVTs were tested performing the two-step calibration procedure. In detail, for each step, 100 measurements of ε, Δφ, and $k_{a}$ were collected with the rated voltage and the rated frequency applied to the LPVTs (room temperature 24 °C). Figure 7 and Figure 8 depict the comparison of ε and Δφ in the absence of an external electric field (step 1) and in its presence (step 2). Once again, as done for the frequencies, two new colors were assigned to the two steps.

A general comment looking at Figure 7, is that the presence of an external field causes an increase of ε. The amount of such an increase varies from one LPVT to another; in particular, the capacitive one (B) is the most affected LPVT.

As for Δφ, the effect of an electric field influencing an LPVT is not appreciable for A and C, while a slight increase is reported for LPVT A.

Turning to more quantitative and deep considerations, for all the LPVTs, the limits fixed by the accuracy class 0.5 did not exceed for either ε or Δφ. Furthermore, the final word on the test results was given by the application of Equations (3) and (4) to the obtained results. Therefore, k and $\varphi_{12}$ were computed and collected in Table 2 together with the limits $\frac{1}{5}\varepsilon_{r}$ and $\frac{1}{3}\Delta \varphi_{r}$.

Table 2 shows that (i) the indexes introduced in Equations (3) and (4) better appreciate the influence of the external fields; (ii) among three LPVTs only B slightly exceeded the limit defined in Equation (3) for k. This is stated after evaluating the uncertainty associated to k for the three LPVTs, which are $3\times 10^{-4}$, $4\times 10^{-4}$, and $3\times 10^{-4}$% for A, B, and C, respectively. Such an uncertainty is obtained from the repeated measurements (100) performed for $k_{a}$, hence exploiting the method A for the evaluation of the uncertainty as defined in the Guide for the Expression of Uncertainty in Measurements (GUM) [46]. Method B used together with method A to obtain the combined uncertainty associated to a measurement was not included due to the fact that the measurands involved in Equation (3) were evaluated with the same devices. Therefore, considering that Equation (3) involves the subtraction of the measurands, the contribution of the reference instrument (if any) does not affect the overall uncertainty.

#### 4.3.3. Tests vs. Temperature

This test consisted of replicating the thermal cycle defined by the calibration procedure in Figure 3. The operating conditions are rated voltage, rated frequency, maximum temperature of 40 °C, and minimum temperature of 5 °C. The maximum temperature was in accordance with the temperature category of the LPVTs, which for all of them was −5 °C to 40 °C. The choice of changing the minimum temperature from −5 °C to 5 °C was taken for three main reasons:

1. Technical limits of the thermostatic chamber adopted;
2. Difficulties in the market to find thermostatic/climatic chambers with sufficient space to contain the MV equipment and to guarantee the distances for the electrical safety;
3. A test performed at 5 °C, which is the average winter temperature in Italy [47], would have provided useful information: (i) what happens to Italian LPVTs working at their lowest average temperature; (ii) if anomalous behavior is reported at 5 °C, then at −5 °C the situation is even worse.

In light of this, the three temperatures adopted to perform the test illustrated in Figure 3 were 5, 24, and 40 °C. For each of the eleven measurement points highlighted in the picture, 100 measurements of ε and Δφ were collected and evaluated. As for the thermal constant τ used in Figure 3, the manufacturers of the three tested LPVTs provided similar values that range between 45 to 60 min. Therefore, to fulfill the requirements of the calibration procedure, 60 min was adopted as an overall value for τ. 

**Figure 9:**
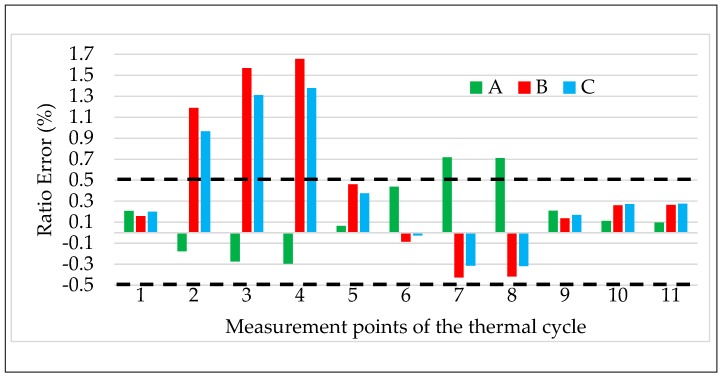
Ratio error vs. temperature for the three LPVTs under test.

**Figure 10:**
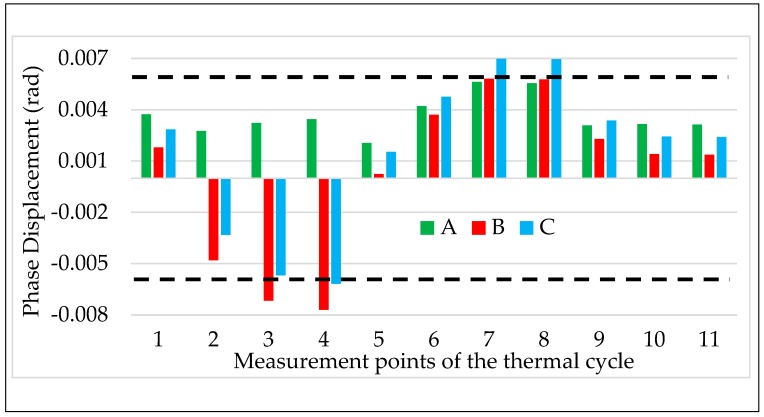
Phase displacement vs. temperature for the three LPVTs under test.

The obtained results are shown in Figure 9 and Figure 10 for ε and Δφ, respectively. According to the previous test results, the ε and Δφ values reported in the graphs are the mean of the 100 values collected during the test.

The two graphs contain three colored bars for each measurement point from #1 to #11. Each bar corresponds to one LPVT from A to C, green, red, and light blue, respectively. Finally, to better assess the results, dashed lines are included in each graph corresponding to the limits of ε and Δφ fixed by the accuracy class (which is 0.5) of the LPVTs under test.

A first general comment on both graphs is that ε and Δφ strictly follow the temperature behavior as depicted in Figure 3, this means, before detailing the obtained results, that LPVTs are quite affected by air temperature, hence their accuracy parameters.

A second general comment is on the direction of the ε and Δφ variation for the three LPVTs. In fact, A exhibits an opposite behavior compared to that of B and C, showing that when ε and Δφ of A experience an increase/decrease, the corresponding values of B and C experience a decrease/increase.

In Figure 9 and Figure 10, point number 2 corresponds to a measurement performed immediately after the air temperature reached 40 °C. In this condition, ε and Δφ already experienced an important variation. In particular, for LPVTs B and C ε increases, exceeding up to two times the 0.5% limit fixed by the accuracy class. LPVT A instead, maintains the same absolute value but experiences a change in sign (from 0.15 to –0.15). Such behavior, as anticipated before, is described by a change in the output of the LPVTs that was used to apply Equation (1). In fact, while $U_{s}$ increases for B and C, it decreases for the LPVT A. However, such behavior is plausible considering that the three LPVTs under test are made from different manufacturers. As for Δφ, again B and C are really affected by the temperature change, while A undergoes just a slight variation from the initial value at 24 °C.

What was reported for point 2 is strengthened by the results of points 3 and 4 where the temperature of 40 °C was maintained for 1.5τ and 3τ, respectively (with τ=60 min). At point 4, ε of B and C exceeded 1.3% (almost three times the limit), while for A, ε was still within the limit. Looking at Figure 10, at point 4, the results are also the same for Δφ. B and C also exceeded the limits for Δφ, while Δφ of A remained within the limits.

From the measurement points 5 to 8 the temperature decreased and passed from the ambient temperature (point 5) to the minimum one (kept for points 6 to 8). From both Figure 9 and Figure 10 it can be appreciated that ε and Δφ, for the three LPVTs, have a continuous variation from point 4 to 8. In particular, all accuracy parameters return within the limits when the air temperature decreases to 24 °C; however, such a variation continues with the reduction of the temperature. In the graphs it is shown that already at point 6 all ε values have changed sign with respect to the ones at high temperature conditions. The same behavior is also observed for Δφ with the exception of LPVT A in which phase displacement experiences slight variations around the initial value.

Focusing on the measurement point 8 (5 °C kept for 2τ), it is possible to assess the effect of a persistent low temperature on the LPVTs. From Figure 9 it is concluded that A is highly affected by the low temperature, having an ε exceeding the upper limit of 0.5%. As for B and C, their ε has an opposite sign compared to the one of the high temperatures, but still within the accuracy limits. Similar comments are also drawn for Δφ by looking at Figure 10; in fact, for the three LPVTs Δφ is close to 0.006 rad, exceeding it only by C.

For the measurement points 9 to 11 the temperature is always 24 °C, after being at 5 °C for 2τ. A joint comment arises for both ε and Δφ observing Figure 9 and Figure 10. The common behavior is a return to the ε and Δφ values assumed by the three LPVTs at the beginning of the thermal cycle test. This means that all accuracy parameters evaluated returned within the limits fixed by the accuracy class (0.5% and 0.006 rad) and that the effects of the thermal cycle vanished.

Summarizing, the application of the calibration procedure part that involves the application of one influence quantity to the LPVTs led to the conclusion that:

- As expected, the tested frequency values do not affect the LPVTs’ accuracy;
- The electric field instead, is a more disturbing quantity that influences the capacitive LPVT more, varying its ε up to the allowed limits. However, none of the ε and Δφ of the tested LPVTs exceeded the accuracy limits fixed by the accuracy class.
- The air temperature appears to be a really stressing influence quantity for all the tested LPVTs, no matter the diverse technologies used to manufacture them. Furthermore, the high temperature is critical for LPVTs B and C, while the low temperature is dangerous for A. This resulted in exceeding the accuracy limits in the different conditions mentioned, hence not guaranteeing the correct operation of the devices. Such a conclusion affects both the DSOs and the final costumers, depending on the sign of the ε and Δφ variations. In other words, the sign of the variation can make the DSOs and the customers save or pay money that they do not deserve.

A final comment for this subsection is dedicated to the standard deviation associated to the values reported in the graphs. Each single test performed above the standard deviation of the mean associated to the mean values was $\leq 10^{-4}$% for ε and $\leq 10^{-6}$ rad for Δφ. Such values were at least three orders of magnitude below the ε and Δφ obtained during the tests. Therefore, they were omitted in each subsection but collected here for the sake of clarity.

In the next two paragraphs, the calibration procedure part, which involves more than one influence quantity, is applied to the three LPVTs.

#### 4.3.4. Tests vs. Two Influence Quantities

The frequency and the electric field are the first two influence quantities combined to fulfill the calibration procedure. The test procedures, described in detail for the single influence quantity tests, are not detailed again in what follows, but simply improved if and when necessary.

**Figure 11:**
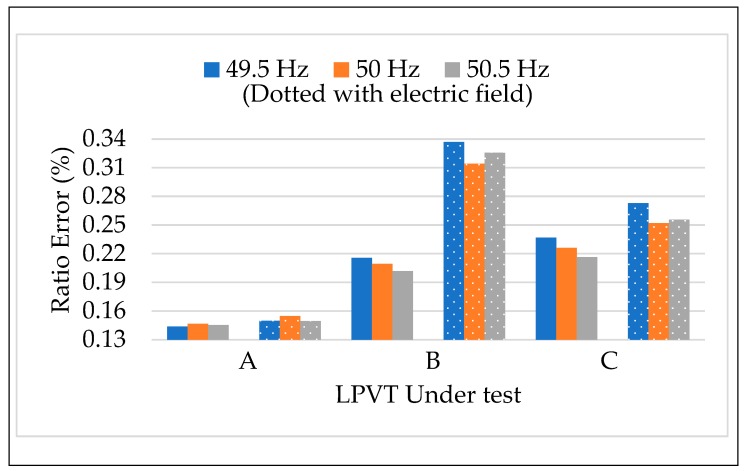
Ratio error vs. frequency + electric field for the three LPVTs under test.

**Figure 12:**
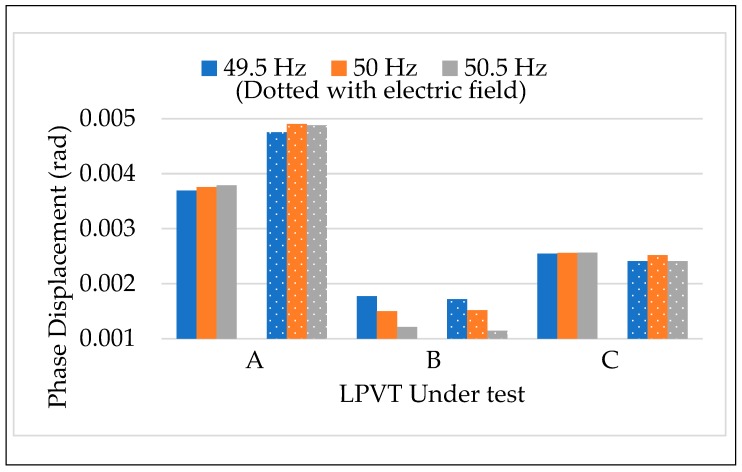
Phase displacement vs frequency + electric field for the three LPVTs under test.

The results of the calibration procedure tests, applying different frequencies along with the external electric field, are depicted in Figure 11 and Figure 12. In both figures, ε and Δφ are presented maintaining the color code introduced for the frequency in Section 4.3.1. In addition, dotted colored bars are used to differentiate the test with or without the presence of an electric field. Therefore, for each LPVT there is two groups of bars: one describing the results without the presence of an electric field (left group), and one with its presence (right group).

A general comment is that what was observed in the results vs. frequency and vs. electric field was confirmed for both ε and Δφ. In particular, the frequency affected neither ε nor Δφ, neither when combined to the presence of an external electric field. Therefore, what emerges from Figure 11 and Figure 12 is that ε and Δφ assume the same values either in the case of LPVTs stressed with an electric field or with electric field plus a frequency different from the rated one. These comments are valid for the three types of tested LPVTs.

**Figure 13:**
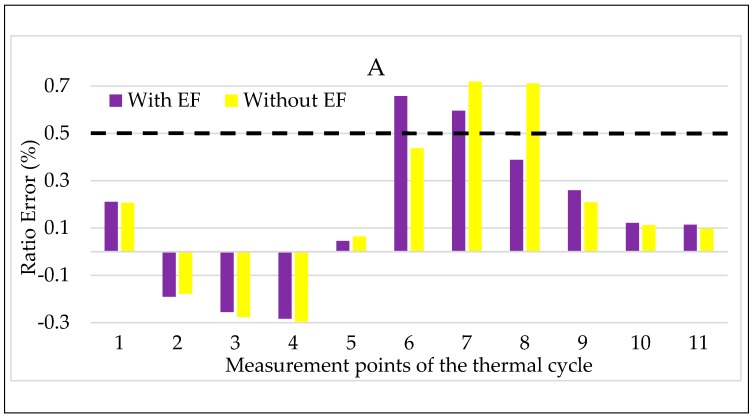
Ratio error vs. temperature + electric field for LPVT A.

**Figure 14:**
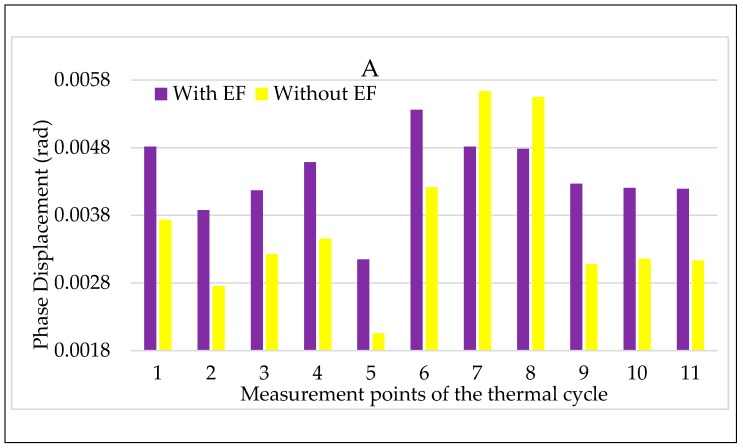
Phase displacement vs temperature + electric field for LPVT A.

Turning to the combination of temperature and electric field, the results for LPVT A are shown in Figure 13 and Figure 14. In both figures, the color code adopted in the tests vs. electric field are maintained: purple for step 2 and yellow for step 2, with and without an electric field, respectively. 

Focusing on Figure 13, the simultaneous presence of temperature and electric field does not seem to affect the LPVT A ratio error. The comment can be extended to all temperatures except for the lowest one, in which the increasing ε, due to the temperature kept for a long period (2τ), is compensated by the effect of the electric field acting on the LPVT. Therefore, the overall result is that, in the long period, the electric field presence makes ε remainwithin the accuracy limits when the temperature is low.

The same comments are not possible for Δφ values shown in Figure 14, from which it is possible to appreciate the negative effect of the combination of electric field and temperature. In particular, the electric field presence worsens the Δφ values, even if they do not exceed the accuracy limits. Another interesting point of Figure 14 is that the beneficial effect of the electric field, highlighted for ε in Figure 13 at a low temperature, is also valid for Δφ.

**Figure 15:**
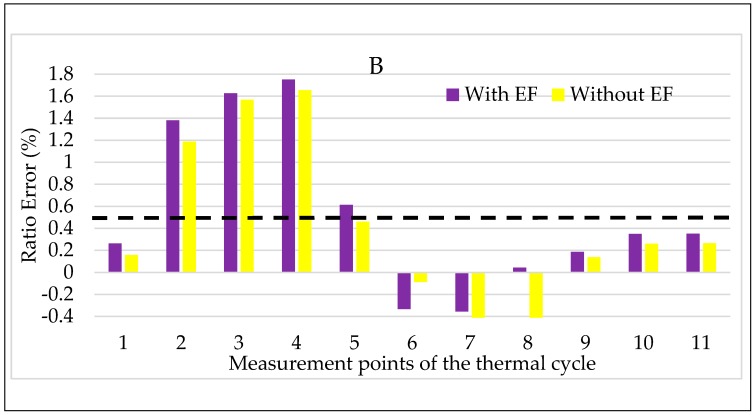
Ratio error vs. temperature + electric field for LPVT B.

**Figure 16:**
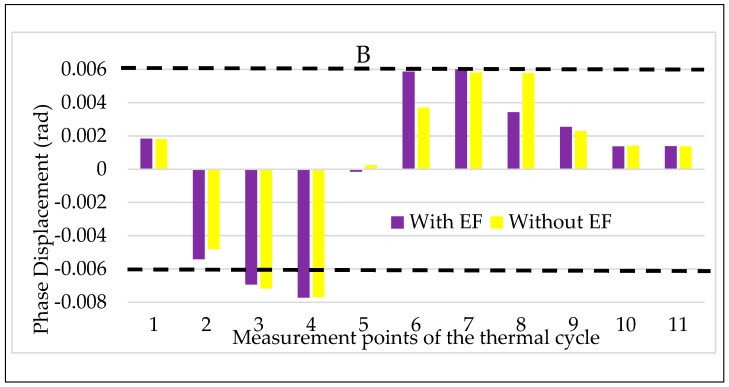
Phase displacement vs temperature + electric field for LPVT B.

With the same notation adopted for A, the results for B are presented in Figure 15 and Figure 16 for ε and Δφ, respectively. Starting from the latter graph, Δφ is almost not affected by the combined presence of temperature and electric field acting on the LPVT. As for ε instead, the general effect is its slight worsening when the electric field is also stressing the LPVT. However, what is worth to highlight from the two figures is the beneficial effect of the electric field when the temperature is low. In accordance to that obtained for LPVT A and B, the reported behavior confirms the “goodness” of the application of an electric field at low temperature, resulting in an improvement of the accuracy parameters of the LPVT.

**Figure 17:**
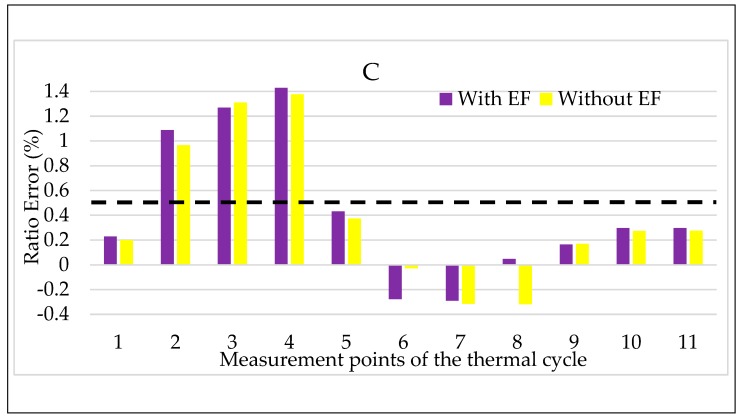
Ratio error vs. temperature + electric field for LPVT C.

**Figure 18:**
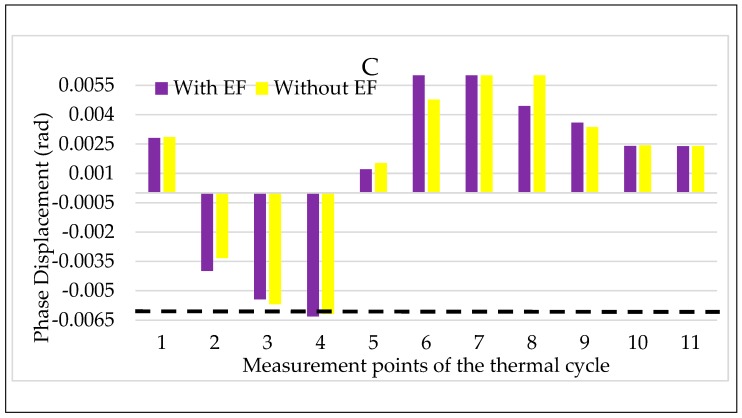
Phase displacement vs. temperature + electric field for LPVT C.

For the sake of completeness, also the graphs for C have been included in Figure 17 and Figure 18. However, the results show the same behavior obtained as for B; therefore, the same comments are not repeated.

**Figure 19:**
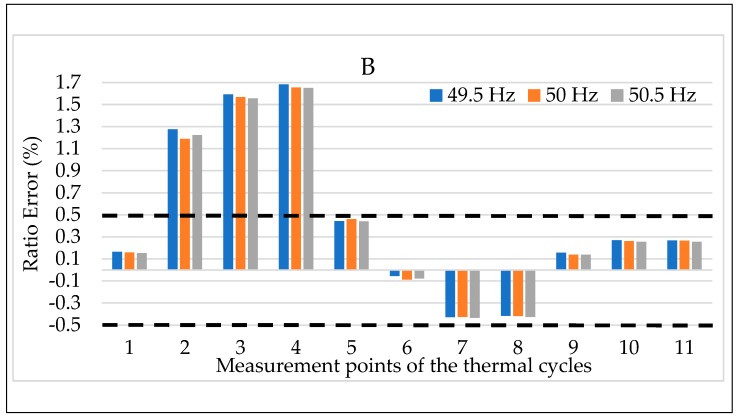
Ratio error vs. temperature + frequency for LPVT B.

**Figure 20:**
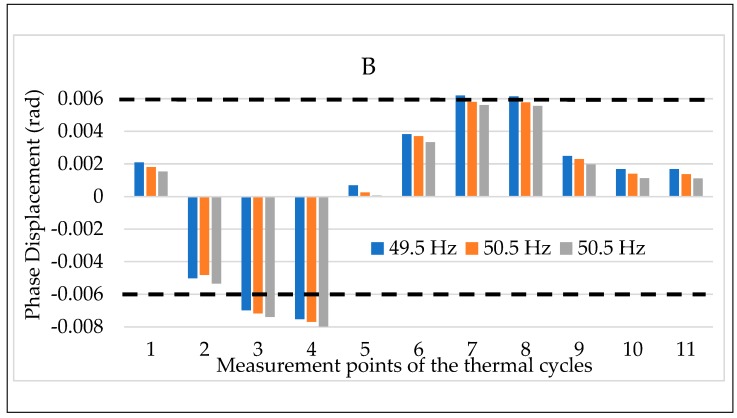
Phase displacement vs. temperature + frequency for LPVT B.

Finally, the results of the temperature plus frequency combined tests are depicted in Figure 19 and Figure 20 for the LPVT B. The choice of presenting B is completely random considering that the behavior represented by the two graphs is valid for all LPVTs. Therefore, ε and Δφ are depicted in Figure 19 and Figure 20, respectively. In detail, for each temperature from #1 to #11, colors blue, orange and grey have been used to indicate the frequencies 49.5, 50, and 50.5 Hz.

As it arises from the two graphs, the frequency variation from its rated value is not affecting the behavior of the LPVT. In other words, when the frequency and the temperature are acting simultaneously on the device, only the latter is significantly changing its behavior, hence its accuracy parameters. These comments are valid for both ε and Δφ.

At the end of the three tests combining two influence quantities at one time it is possible to conclude really interesting information:

- Overall, it was demonstrated in Section 4.3.1 that the frequency does not significantly affect the performance of the LPVTs; this behavior is confirmed also when such quantity is combined either with electric field or temperature (and for both ε and Δφ). In fact, these last two influence quantities are far more critical than the frequency;
- Temperature has emerged as the main critical quantity. However, its combination with the electric field resulted in two main behaviors. One, where ε and Δφ worsened or were not affected at all at ambient or high temperatures. Another, where ε and Δφ improved by the presence of an electric field superimposed to the temperature effect, at low temperature. Furthermore, the beneficial effect of the electric field at 5 °C resulted in ε returned within the accuracy limits when they exceeded the effect of the temperature.

In addition, for the tests involving two influence quantities, the standard deviations of the mean associated to ε and Δφ, for the three LPVTs, were $\leq 10^{-4}$% and $\leq 10^{-6}$ rad, respectively; far below the measured quantities and their assessed variations.

The first conclusion of these test series is that a calibration procedure involving more than one influence quantity is helpful to find the overall effect on the LPVT under test. Such an effect is not always a worsening of the accuracy parameters of an LPVT, due to the simultaneous presence of influence quantities.

#### 4.3.5. Tests vs. All Three Influence Quantities

The calibration procedure ends with testing the off-the-shelf LPVTs while simultaneously applying the three influence quantities described in the procedure: temperature, frequency, and external electric field. For this set of tests, the results presented are the mean of 100 values of ε and Δφ.

**Figure 21:**
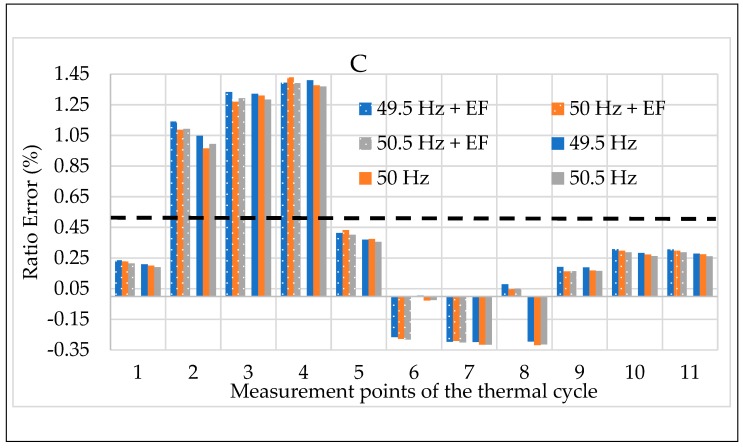
Ratio error vs. the three influence quantities for LPVT C.

**Figure 22:**
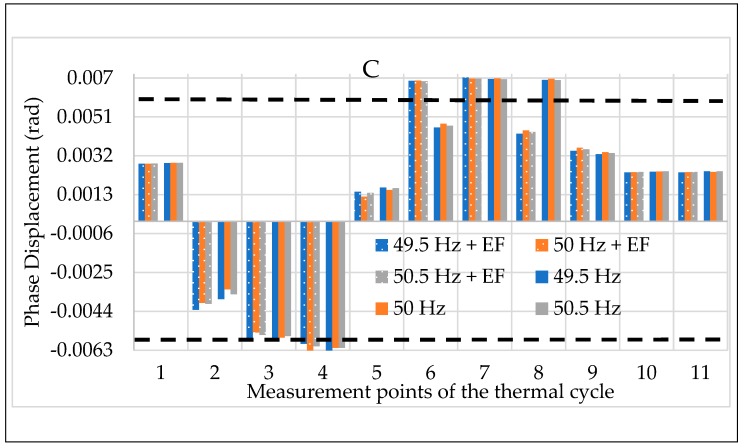
Phase displacement vs. the three influence quantities for LPVT C.

The results depicted in Figure 21 and Figure 22 refer to ε and Δφ for LPVT C, respectively. The two figures were designed in accordance with the notations implemented in the previous graphs of the manuscript. In detail, the color code for the frequencies and the dotted bars for the electric field were kept in these graphs to improve their readability. Therefore, for each measurement point of the thermal cycles there are two groups of three bars: one for the results without any electric field affecting the LPVT (right group) and one for the results with the electric field (left group). In each group of bars, the three values represent the three frequencies tested.

Again, among the three LPVTs, C was randomly chosen because the depicted behavior was confirmed for all the tested LPVTs (and for both ε and Δφ). In particular, from Figure 21 and Figure 22 it can be highlighted that even in the case of three influence quantities acting together on the LPVTs, the frequency effect is not significant on their accuracy. Furthermore, this last set of tests confirmed what was obtained in the tests with the combination between temperature and electric field: the sum of two disturbing effects (or external influence factors) does not always result in a worse case for the LPVT under test. On the contrary, what arises from all the tests is that sometimes the combination of multiple influence quantities may lead to beneficial effects on the LPVTs’ accuracy.

## 5. Conclusions

This paper aims at proposing a new calibration procedure focusing on more realistic test conditions for the tested devices, the low-power voltage transformers. The presented tests combine the presence of frequency, electric field, and temperature before assessing the accuracy parameters of the transformer (in terms of ratio error and phase displacement). Afterwards the calibration procedure was immediately implemented on a case study involving three off-the-shelf low-power voltage transformers of different types and manufacturers. The results indicate that it is significant to test the devices in more realistic conditions; such tests appreciate behaviors different from the expected and from the ones obtained from testing the low-power voltage transformers by applying one influence quantity at the time. In the particular case of the case study, for example, it was highlighted that the combined presence of low temperatures and electric field had a beneficial effect on the accuracy parameters of the transformers. Furthermore, it demonstrated how temperature differently affects the transformers under test. Such examples do not pretend to state that all transformers of the same kind behave at the same way, but confirm the need to fill in the gaps in the tests provided by the Standards and to make them as realistic as possible.

## Figures and Tables

**Table 1:** Main characteristics of the three LPVTs under test. AC, accuracy class.

LPVT	Type	$V_{1R}\;(V)$	$V_{2R}\;(V)$	AC
A	Resistive	$20000/\sqrt{3}$	$3.25/\sqrt{3}$	0.5
B	Capacitive	$20000/\sqrt{3}$	1.35	0.5
C	Active	$20000/\sqrt{3}$	1	0.5

**Table 2:** Results of the application of Equations (3) and (4) for the test vs. electric field.

LPVT	k [%]	$\frac{1}{5}\varepsilon_{r}\;(\%)$	$\varphi_{12}\;(\mathrm{rad})$	$\frac{1}{3}\Delta \varphi_{r}\;(\mathrm{rad})$
A	−0.0083	0.1	−0.001	0.002
B	−0.1047	0.1	0.000	0.002
C	−0.0258	0.1	0.000	0.002
